# Transnasal Humidified Rapid Insufflation Ventilatory Exchange (THRIVE) for the Operative Management of Retrograde Cricopharyngeus Dysfunction

**DOI:** 10.1002/oto2.70173

**Published:** 2025-10-23

**Authors:** Amy B. Leming, Dylan G. Vance, Andrew G. Tritter, Zao Mike Yang

**Affiliations:** ^1^ Department of Otorhinolaryngology–Head and Neck Surgery UTHealth Houston‐McGovern Medical School Houston Texas USA; ^2^ Department of Otolaryngology–Head and Neck Surgery UT Health San Antonio San Antonio Texas USA

**Keywords:** apneic oxygenation, esophagus, high‐flow nasal oxygen, RCPD, THRIVE

## Abstract

**Objective:**

Transnasal Humidified Rapid Insufflation Ventilatory Exchange (THRIVE) is a method of apneic oxygenation gaining popularity in upper aerodigestive surgery. Retrograde cricopharyngeus muscle dysfunction (RCPD) is characterized by the inability to belch, managed by intraoperative injection of botulinum toxin to the cricopharyngeus muscle (CPBI), often performed under general anesthesia with endotracheal intubation. We sought to assess the safety and efficacy of THRIVE when performing CPBI for RCPD.

**Study Design:**

We conducted a retrospective review of adult RCPD patients undergoing CPBI under general anesthesia with THRIVE.

**Setting:**

The study was conducted at both the University of Texas Health Science Centers in Houston and San Antonio over a 5‐month period from June 2023 to November 2023.

**Methods:**

Patients were placed under general anesthesia using THRIVE. CPBI was performed. Demographic, clinical, and anesthesiologic data were collected and analyzed.

**Results:**

In total, 32/39 (82%) were able to maintain oxygenation throughout the procedure. Mean (standard deviation) time from induction to paralytic reversal was 7.8 (3.3) minutes. Time from induction to return of spontaneous breathing was 9.9 (3.2) minutes. Excluding seven patients who required “rescue” bag‐mask ventilation due to failure to maintain oxygenation, the median oxygen saturation nadir was 97.7% (range 92%‐100%). The average increase in end‐tidal CO_2_ level (EtCO_2_) was 1.14 mm Hg/min. Body mass index (BMI) significantly predicted failure to maintain oxygenation on binary logistic regression (coefficient 0.239, *P* = .010).

**Conclusion:**

THRIVE is a feasible means of apneic oxygenation when performing operative CPBI for patients with RCPD, although the need for “rescue” ventilation may occur at a higher rate in comparison to existing literature for laryngotracheal surgery.

**Level of Evidence:**

IV.

Transnasal Humidified Rapid Insufflation Ventilatory Exchange (THRIVE) is a method of apneic oxygenation that has recently gained popularity in upper aerodigestive surgery since it was first described by Patel and Nouraei in 2015.^1^ This technique delivers continuous, warm, humidified oxygen at a high flow rate via nasal cannula to apneic patients. Continuous oxygen insufflation causes denitrogenation of the anatomical dead space and supports lung gas exchange through a phenomenon called aventilatory mass flow. Some ventilation is produced as the inspired oxygen flow flushes out expired carbon dioxide, reducing the rate of carbon dioxide accumulation and allowing the extended use of THRIVE with a reduced risk of hypercapnia in an otherwise apneic patient. Additionally, atelectasis is prevented through some degree of positive airway pressure, and desiccation of the nasal and pharyngeal mucosa is reduced with humidification, maintaining both mucociliary clearance and patient comfort.^1^, ^2^

While high‐flow oxygen delivery has been previously applied in intensive care units, respiratory medicine, and in anesthesia for preoxygenation, apneic oxygenation solely using THRIVE in an operative setting has only recently been utilized.^2^, ^3^ Benefits of THRIVE are substantial, including improved visualization in an otherwise crowded operative field, decreased risk of barotrauma related to jet or mechanical ventilation, prevention of oropharyngeal or laryngeal injury, and reduction in the incidence of reflux and microaspiration.^4^, ^5^, ^6^ Currently, there exists a moderate body of literature regarding successful application during upper airway procedures including laryngeal microsurgery, panendoscopy with biopsy, dilation of subglottic stenosis, tracheostomy, or even tracheal resection.^2^, ^6^, ^7^, ^8^, ^9^, ^10^ Fewer reports have evaluated the safety and efficacy of THRIVE during primarily esophageal procedures.^5^, ^11^ Even so, preliminary data have demonstrated that THRIVE appears to be a feasible means of apneic oxygenation for patients undergoing endoscopic esophageal surgery under general anesthesia in lieu of endotracheal intubation.^11^

Retrograde cricopharyngeus muscle dysfunction (RCPD) is a disorder characterized primarily by the inability to belch, also known as “eructile dysfunction” or “abelchia,” along with several common symptoms stemming from this inability. Although first described in a case report from 1987 as dysfunction of the upper esophageal sphincter (UES), the disorder was not formally named or given widespread recognition until a seminal publication from Bastian and Smithson in 2019. Therein, the authors describe the cardinal symptoms of RCPD, which, in addition to the inability to belch, include abdominal fullness and/or bloating; gurgling noises in the chest or lower neck; excessive flatulence in the absence of any concurrent gastrointestinal or oropharyngeal pathology; and social inhibition stemming from these symptoms.^12^, ^13^ This symptomatology is hypothesized to occur due to the failure of UES relaxation during periods of esophageal distension, thus preventing the normal belch reflex from occurring.^14^ The current gold standard of treatment, which is also used for diagnostic confirmation, is the injection of botulinum toxin into the cricopharyngeus muscle (CPBI). While the injection may be performed in the office using a percutaneous/transcervical approach under EMG guidance, many physicians and patients prefer the operative endoscopic approach in the operating room, primarily due to its significantly higher success rate.^15^

Given the increased recognition of THRIVE as a valuable tool for airway management in surgical procedures of the upper aerodigestive tract, we sought to assess the safety and efficacy of THRIVE when performing CPBI for those patients with RCPD.

## Methods

We conducted a retrospective review of prospectively collected data on adult RCPD patients undergoing CPBI under general anesthesia with THRIVE for airway management. This included patients treated at the University of Texas Health Science Centers in Houston and San Antonio over a 5‐month period from June 2023 to November 2023. The study was approved by the University of Texas Health System Institutional Review Board (IRB).

Demographic and clinical data were collected. For these procedures, patients were placed under general anesthesia using THRIVE without endotracheal intubation. Induction of anesthesia and subsequent airway management were performed in accordance with the protocol described by Yang et al.^11^ Patients were preoxygenated while awake with a high‐flow nasal cannula running at 30 to 50 liters per minute (LPM). After induction of anesthesia with intravenous sedative (propofol) and a long‐acting neuromuscular blocking agent (rocuronium), the nasal cannula was briefly lowered, and a mask was used to measure and record the pre‐procedure end‐tidal CO_2_ level (EtCO_2_). EtCO_2_ was measured by taking the average from a series of three mask‐delivered breaths at 100% FiO_2_, and the nasal cannula was replaced. THRIVE was then initiated with an increase in oxygen flow to 70 LPM through a humidified nasal cannula. Patients were maintained under total intravenous anesthesia (TIVA) with paralysis until the end of the procedure, at which point neuromuscular blockade was reversed with sugammadex, and a mask was again used to check EtCO_2_ via an average of three breaths before allowing the patient to spontaneously wake up on their high‐flow nasal cannula. Time points from induction to reversal of paralytic and return of spontaneous breathing were recorded. Oxygen saturation (SpO_2_) nadir was also recorded, and note was made of any patients who required “rescue” mask ventilation during their procedure due to failure to maintain oxygenation.

For the surgical procedure itself, a Slimline (Holinger‐Benjamin or Dohlman) diverticuloscope was used for exposure of the cricopharyngeus (CP) muscle. For patients whose anatomy limited the access of this instrument, a Dedo or Garrett‐Ossoff‐Pilling laryngoscope was instead used for exposure. Following inspection of the cervical esophagus with a zero‐degree telescope, a total of 75 to 85 units of onabotulinum toxin A at a dilution of 10 units/0.1 mL was then injected into the body of the muscle across three separate sites (right, left, and midline).

Continuous variables are presented as mean (standard deviation) for parametric data. Median was used to represent SpO_2_. The Pearson correlation coefficient was calculated to determine the correlation between clinical data and tolerance of THRIVE. Linear regression was used for numeric variables. Statistical significance was determined using a level of *α* = .05. Statistical analysis was performed in Excel and subsequently finalized in ChatGPT.

## Results

**Figure 1:**
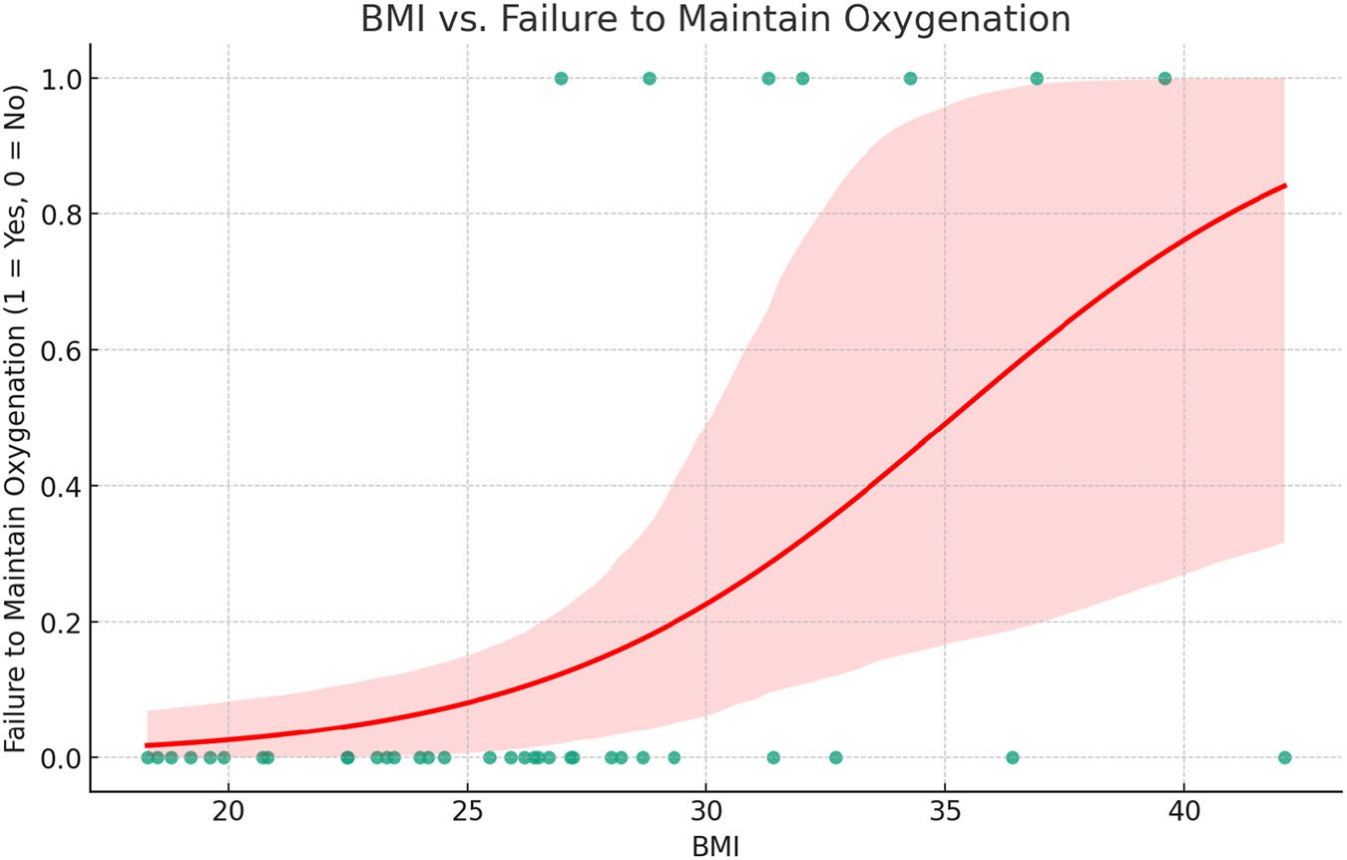
Binary logistic regression of body mass index (BMI) compared to failure to maintain oxygenation (Y/N). Coefficient 0.239, *P* = .010. Pseudo *R* ^2^ value is 0.2561.

In total, 39 cases performed from June 2023 to November 2023 met the inclusion criteria. In total, 32/39 (82%) were able to maintain oxygenation throughout the entirety of their procedure. Average age in years was 30.4 (±11.4), 25 (64%) were female, and average Body mass index (BMI) was 26.7 (±5.8) (Table 1). All patients were in a state of good medical health, with no patients having a Charleston Comorbidity Index score higher than 2. Mean apneic time (from induction to return to spontaneous breathing) was 9.9 (±3.2) minutes. Mean time from induction to reversal was 7.8 (±3.3) minutes. Excluding the seven patients who required “rescue” bag‐mask ventilation due to failure to maintain oxygenation, median SpO_2_ nadir for the procedure was 97.7% (range 92%‐100%), while the median for those who failed to maintain oxygenation was 85.5% (range 77%‐90%). Average increase in EtCO_2_ from induction to reversal of anesthesia was 1.14 mm Hg/min. BMI significantly predicted failure to maintain oxygenation on binary logistic regression (coefficient 0.239, *P* = .010) (Figure 1). Age and gender did not show a significant correlation with failure to maintain oxygenation. Patients with a BMI of more than 30 had a failure rate of 55.5% with THRIVE. Those with a BMI of 25 to 30 had a 15.5% failure rate, while those with a BMI less than 25 did not have any oxygenation failures in our cohort (Table 2).

**Table 1:** Patient Demographics Including Age, Body Mass Index (BMI), and Gender

Patient demographics	
Age (mean ± SD), y	30.4 (11.4)
Body mass index (mean ± SD)	26.7 (5.85)
Gender	25 (64%) female

**Table 2:** Body Mass Index (BMI) and Failure Rate^a^

BMI and failure rate	
BMI less than 25	0
BMI 25‐30	2 (15.5%)
BMI greater than 30	5 (55.5%)

^a^ The number of patients who failed to maintain oxygenation with Transnasal Humidified Rapid Insufflation Ventilatory Exchange (THRIVE) in respective BMI cohorts.

Excluding the five patients who did not follow up after their procedure, 94.5% of patients reported at least partial if not complete improvement in their original RCPD symptoms at a 6‐week postoperative visit. The remaining 5.5% had no improvement.

## Discussion

THRIVE has been demonstrated to be an effective means of airway management during microlaryngeal and endoscopic airway procedures.^2^, ^5^, ^6^, ^7^, ^8^, ^9^, ^10^, ^16^, ^17^, ^18^ Because it obviates the need for endotracheal intubation, operative and anesthesia time^17^ as well as postoperative pain^18^ may be decreased. Although no studies have specifically evaluated the average operative length for CPBI, many institutions openly quote approximately 15 to 30 minutes for the procedure in online patient care documents. In our cohort, the average total time was only 9.9 minutes, and most patients (82%) were able to successfully maintain oxygenation with THRIVE throughout the entirety of their procedure without use of “rescue” ventilation. A recent literature review by Ananthapadmanabhan et al noted a correlation between failure to maintain oxygenation with THRIVE and prolonged procedure time. While THRIVE has been shown to decrease the rate of development of hypercapnia in otherwise apneic patients, respiratory acidosis will inevitably develop as the procedure proceeds, requiring discontinuation of THRIVE.^19^ While this study was aimed at evaluating a very specific patient population and procedure, THRIVE may represent a preferred method of airway management in patients undergoing relatively short, uncomplicated esophageal procedures.

The present study data demonstrate that even with the limited time constraints under which THRIVE may be utilized, procedural efficacy is not adversely affected. At 6 weeks postoperatively, 34 out of 39 patients presented for their follow‐up appointment, 94.5% of which reporting a significant response to treatment. Similarly, in the largest case series to date describing long‐term CPBI outcomes, Hoesli et al. reported major relief of symptoms in 95% of patients within 4 weeks of surgery.^20^ When compared with traditional intubation as performed in the aforementioned study, utilization of THRIVE at our institution yielded almost identical results.

At rest, the UES is closed due to tonic contraction of the CP muscle and collapse of the pharyngoesophageal segment even during operative neuromuscular paralysis. To ensure accurate injection placement in the anesthetized patient, visualization of the CP is best achieved with a rigid endoscope that can distend the UES and fully expose the muscle. Distention of the UES with a rigid endoscope has been theorized to potentially alter airflow dynamics with the use of THRIVE, redirecting the continuous flow of oxygen away from the airway and into the esophagus.^11^ However, preliminary reports have shown that THRIVE is a reasonable means of apneic oxygenation for patients undergoing a variety of short esophageal procedures with similar apneic times to those reported in laryngotracheal procedures.^5^, ^11^

In the present study, EtCO_2_ was directly associated with the duration of apnea. Linear regression models suggest that EtCO_2_ increases at a rate of approximately 1.14 mm Hg/min or 0.152 kPa/min while under THRIVE for CPBI. Similar studies describe comparable values, including one report of endoscopic esophageal intervention and several reports regarding laryngotracheal intervention (0.127 kPa/min and 0.11‐0.17 kPa/min, respectively).^1^, ^11^, ^21^, ^22^ These values are well below the rate of rise (0.35‐0.45 kPa/min) previously reported in apneic patients with no supplemental oxygenation, showing that THRIVE may reduce the deleterious effects of apneic hypercapnia.^23^ The total time from induction to return of spontaneous breathing in all our cases was less than 20 minutes, indicating that successful CPBI is easily achievable with THRIVE utilization.

While the potential redirection of airflow caused by rigid distention of the UES is typically minimal enough to provide adequate oxygenation throughout a short procedure, this alteration of dynamics may disproportionately affect patients with a higher BMI. Current literature surrounding the use of THRIVE in both esophageal and laryngotracheal procedures indicates that BMI may be a potential prognostic indicator for its failure.^5^, ^11^, ^21^ Our data are consistent with these findings as BMI significantly predicted failure to maintain oxygenation. Over half (55.5%) of patients with a BMI more than 30 in our cohort required “rescue” bag‐mask ventilation, whereas no patients with a BMI under 25 failed to maintain oxygenation with THRIVE. A recent literature review by Ananthapadmanabhan et al noted that reduced functional residual capacity (FRC) may result in shortened apneic windows and subsequent THRIVE failure. Medical conditions that affect gas exchange, cardiac output, oxygen carrying capacity, or increased oxygen consumption may contribute to faster desaturation.^19^ Patients with underlying cardiopulmonary disease, anemia, obesity, or even pregnancy require further attention before consideration for THRIVE as a means of oxygenation in upper aerodigestive tract procedures.

While not specifically evaluated in our study, we postulate that the choice of a rigid endoscope may play a role in success as well. CPBI can be successfully performed with a variety of rigid laryngoscopes, diverticuloscopes, and esophagoscopes, where each affords particular advantages for exposure in a wide range of patients.^12^ However, in those with an already crowded pharynx, any additional obstruction of the laryngotracheal airway added by suspension with rigid instrumentation may result in further restriction of normal airflow dynamics and resultant desaturation. Such crowding may itself be worsened by obesity, further helping to explain our findings of decreased THRIVE tolerance in patients with an elevated BMI. The authors believe that this may particularly be the case with the Holinger‐Benjamin (Dohlman) diverticuloscope, which provides excellent distension and exposure of the CP muscle, but also has a wider shape than most other rigid endoscopes with more potential for airflow obstruction. Finally, inadvertent catching of the epiglottis by the rigid endoscope can cause it to retroflex during canalization of the cervical esophagus, potentially sealing off the laryngeal vestibule and further limiting diffusion of oxygen to the distal airway.

Although most reports published so far describe a total dose range between 50 and 100 units of onabotulinum toxin injected into several sites of the bilateral CP muscle, the ideal dosage was not specifically evaluated in this study. The authors chose a dose range of 75 to 85 units to maximize use of the full 100‐unit vial while accounting for product loss in the dead space along the 22 cm barrel of the orotracheal injector that was used.

As with laryngotracheal procedures, THRIVE provides improved visualization of critical structures when utilized for CPBI. Canalization of the esophagus can be much easier in the absence of an endotracheal tube that would otherwise occupy space in the pharynx. Additionally, as described by Bastian and Smithson, a small subset of patients may develop recurrent symptoms requiring subsequent procedures.^12^ Use of THRIVE avoids airway trauma traditionally related to intubation, especially in a short elective procedure such as CPBI. This may particularly benefit RCPD patients, a cohort that is often exceptionally attuned to their upper aerodigestive symptoms. Although not directly compared to intubated patients in this study, the authors noted suitable operative access and visualization to perform successful CPBI in patients oxygenated with THRIVE.

Given the specific patient population, limitations of this study include its relatively small sample size and lack of a randomized comparison with intubated patients. Additionally, despite our short overall operative length, the average rate of EtCO_2_ rise was slightly higher than other studies looking at THRIVE in esophageal procedures.^11^ Factors not specifically studied, which may have affected this outcome, include variability in successful exposure between resident and attending physicians and smoking status in specific patients. As such, additional studies are needed to evaluate the utilization of THRIVE in patients undergoing other endoscopic esophageal procedures under general anesthesia.

## Conclusion

THRIVE is a feasible means of apneic oxygenation when performing CPBI in the operating room for patients with RCPD, although the need for “rescue” ventilation occurred at a higher rate in comparison to existing literature for laryngotracheal surgery. Caution should be taken when using THRIVE to perform CPBI on patients with an elevated BMI, as this was predictive of failure to maintain adequate oxygenation.

## Author Contributions

**Amy B. Leming**, design, analysis, drafting and revision, and presentation of research; **Dylan G. Vance**, design, analysis, and presentation of research; **Andrew G. Tritter**, conduct, analysis, drafting, and revision; **Zao Mike Yang**, conduct.

## Disclosures

### Competing interests

The authors declare no conflicts of interest.

### Funding source

The authors received no financial support for the research, authorship, or publication of this article.
