# Correction to “Transnasal Humidified Rapid Insufflation Ventilatory Exchange (THRIVE) for the Operative Management of Retrograde Cricopharyngeus Dysfunction”

**DOI:** 10.1002/oto2.70196

**Published:** 2026-01-19

Leming, A.B., Vance, D.G., Tritter, A.G. and Yang, Z.M. (2025), Transnasal Humidified Rapid Insufflation Ventilatory Exchange (THRIVE) for the Operative Management of Retrograde Cricopharyngeus Dysfunction. OTO Open, 9:e70173. https://doi.org/10.1002/oto2.70173

The author list was mistakenly published in the incorrect order. The designated corresponding author was also incorrect. These have been modified to the correct order and designation, respectively. The revised author order is Amy B. Leming MD, Dylan G. Vance MD, Zao Mike Yang MD, and Andrew G. Tritter MD. The corresponding author is:

Andrew G. Tritter MD

Department of Otorhinolaryngology

UTHealth Houston ‐ McGovern Medical School 6431 Fannin St, MSB 5.036

Houston, Texas, USA

Email: Andrew.g.tritter@uth.tmc.edu

We apologize for this error.

